# Attention-Deficit/Hyperactivity Disorder Medications and Work Disability and Mental Health Outcomes

**DOI:** 10.1001/jamanetworkopen.2024.2859

**Published:** 2024-03-20

**Authors:** Heidi Taipale, Jakob Bergström, Katalin Gèmes, Antti Tanskanen, Lisa Ekselius, Ellenor Mittendorfer-Rutz, Magnus Helgesson

**Affiliations:** 1 Division of Insurance Medicine, Department of Clinical Neuroscience, Karolinska Institutet, Stockholm, Sweden; 2 Niuvanniemi Hospital, Kuopio, Finland; 3 School of Pharmacy, University of Eastern Finland, Kuopio, Finland; 4 Department of Women’s and Children’s Health, Uppsala University, Uppsala, Sweden; 5 Department of Public Health and Caring Sciences, Health Equity and Working Life, Uppsala University, Uppsala, Sweden

## Abstract

**Importance:**

Individuals with attention-deficit/hyperactivity disorder (ADHD) often have comorbid psychiatric conditions. Relatively little is known about how specific ADHD medications are associated with overall treatment outcomes among these patients.

**Objective:**

To investigate the association of the use of specific ADHD medications with hospitalization outcomes and work disability among adolescents and adults with ADHD.

**Design, Setting, and Participants:**

This nationwide register-based cohort study identified individuals (aged 16-65 years) with ADHD from Swedish nationwide registers of inpatient health care, specialized outpatient health care, sickness absence, and disability pension during the years 2006 to 2021. Data analysis was performed from November 2022 to August 2023.

**Exposure:**

Use of specific ADHD medications.

**Main Outcomes and Measures:**

The main outcome measure was psychiatric hospitalization, and secondary outcomes were suicide attempt and/or death by suicide, nonpsychiatric hospitalization, and work disability (ie, sickness absence or disability pension). The risk of outcomes between use vs nonuse periods of ADHD medications was compared in a within-individual design, where a person acts as their own control, and was analyzed with stratified Cox models.

**Results:**

A total of 221 714 persons with ADHD were included in the study cohort (mean [SD] age, 25.0 [11.2] years; 120 968 male individuals [54.6%]). Methylphenidate was the most commonly used ADHD medication (151 837 individuals [68.5%]), followed by lisdexamphetamine (78 106 individuals [35.2%]) during the follow-up (mean [SD], 7.0 [4.7] years). The following medications were associated with a decreased risk of psychiatric hospitalization: amphetamine (adjusted hazard ratio [aHR], 0.74; 95% CI, 0.61-0.90), lisdexamphetamine (aHR, 0.80; 95% CI, 0.78-0.82), ADHD drug polytherapy (aHR, 0.85; 95% CI, 0.82-0.88), dexamphetamine (aHR, 0.88; 95% CI, 0.83-0.94), and methylphenidate (aHR, 0.93; 95% CI, 0.92-0.95). No associations were found for modafinil, atomoxetine, clonidine, and guanfacine. Decreased risk of suicidal behavior was associated with the use of dexamphetamine (aHR, 0.69; 95% CI, 0.53-0.89), lisdexamphetamine (aHR, 0.76; 95% CI, 0.68-0.84), and methylphenidate (aHR, 0.92; 95% CI, 0.86-0.98). None of the medications was associated with increased risk of nonpsychiatric hospitalization; instead, use of amphetamine, lisdexamphetamine, polytherapy, dexamphetamine, methylphenidate, and atomoxetine were associated with decreased risk of nonpsychiatric hospitalization. The results regarding work disability were significant only for the use of atomoxetine (aHR, 0.89; 95% CI, 0.82-0.97), especially among adolescents and young adults aged 16 to 29 years, (aHR, 0.82; 95% CI, 0.73-0.92).

**Conclusions and Relevance:**

In this nationwide cohort study of adolescents and adults with ADHD, the use of ADHD medication was associated with fewer hospitalizations for both psychiatric and nonpsychiatric morbidity and lower suicidal behavior.

## Key Points

**Question:**

Are specific attention-deficit/hyperactivity disorder (ADHD) medications associated with the risk of hospitalization outcomes and work disability among adolescents and adults with ADHD?

**Findings:**

In this Swedish nationwide cohort study of 221 714 individuals with ADHD, lisdexamphetamine and other amphetamine derivatives were associated with decreased risk of psychiatric hospitalization, nonpsychiatric hospitalization, and suicide attempts and/or death by suicide.

**Meaning:**

These findings suggest that the use of ADHD medication is associated with less mental health morbidity in adolescents and adults with ADHD.

## Teaser

This cohort study investigates the association of specific attention-deficit/hyperactivity disorder (ADHD) medications with hospitalization outcomes and work disability among Swedish adolescents and adults with ADHD.

## Introduction

Attention-deficit/hyperactivity disorder (ADHD) is a common neurodevelopmental disorder characterized by inattention, hyperactivity-impulsivity, or both.^1^ The prevalence of ADHD has been estimated to be approximately 5%.^2^ Although symptoms start in childhood, they often persist into adulthood, with an estimated ADHD prevalence of 2.5% among adults.^3^ ADHD often co-occurs with other psychiatric disorders, such as communication disorders, intellectual and learning disabilities, autism spectrum disorders, mood disorders, anxiety disorders, and substance use disorders.^1^ Heterogeneity in symptom profiles and psychiatric comorbidities are challenges in the treatment of ADHD in children, adolescents, and adults.

Clinical care guidelines typically recommend pharmacotherapy as a part of the treatment regimen for ADHD, in addition to psychosocial interventions.^4^ Both stimulant and nonstimulant medications are generally efficacious for ADHD symptoms according to a meta-analysis^5^ of short-term, placebo-controlled, randomized clinical trials. When considering both efficacy and safety, that meta-analysis^5^ supported methylphenidate use in children and adolescents and amphetamine use in adults as first-choice medications. In addition to ADHD core symptoms, there is considerable evidence that ADHD medications, especially stimulants, improve functioning and quality of life.^6^ Less is known about the long-term effectiveness and safety of ADHD pharmacotherapies. Previous observational studies have reported that ADHD medication use, mainly regarding stimulants, is associated with several beneficial outcomes, such as decreased risk of suicide attempts,^7^ substance use disorders,^8^ depression,^9^ motor vehicle accidents,^10^ unintentional injuries,^11^ and long-term unemployment.^12^ However, there are also concerns that long-term stimulant use may be associated with adverse outcomes. Because these medications tend to increase blood pressure and heart rate,^13^ they might increase the risk of cardiovascular diseases. An increased risk of seizures,^14^ and possibly an increased risk of psychosis or mania,^15,16^ are also concerns. There is only preliminary and conflicting evidence from small studies on whether ADHD medication improves labor market outcomes, such as work ability.^17,18^

A previous Swedish study^19^ found that the prevalence of any psychiatric comorbidity was 52% among children and adults with ADHD, compared with only 7% among individuals without ADHD, with a lifetime prevalence of up to 92%.^20^ The previous study^19^ also reported that psychiatric comorbidity increased the risk of mortality among individuals with ADHD, especially when the comorbidity was substance use disorder. Because ADHD medications in long-term observational studies are associated with both beneficial psychiatric outcomes, such as decreased risk of depression and suicide, and with possible adverse outcomes such as the risk of psychosis, we aimed to investigate the association of specific ADHD medication use with the risk of psychiatric hospitalization in a nationwide cohort of adolescents and adults with ADHD. In addition, we studied the associations with suicide attempts and/or deaths, hospitalization for nonpsychiatric reasons as a general safety outcome, and work disability, defined as sickness absence and/or disability pension.

## Methods

This cohort study was approved by the Regional Ethical Review Board, Karolinska Institutet, Stockholm, Sweden. According to current Swedish law, the use of registry data for research purposes does not require informed consent from individuals held in these registries. We have complied with the Strengthening the Reporting of Observational Studies in Epidemiology (STROBE) reporting guidelines for cohort studies.

For this study, we included all individuals of working age, 16 to 65 years old, residing in Sweden who had received a diagnosis of ADHD between January 2006 and December 2021. A diagnosis was defined by the *International Statistical Classification of Diseases and Related Health Problems, Tenth Revision (ICD-10)* code F90 according to records in the nationwide registers of inpatient care, specialized outpatient care, sickness absence, and disability pension.

Data for the cohort were linked from several nationwide registers via unique personal identification numbers assigned to all residents at birth or immigration. Inpatient and specialized outpatient care data were collected from the National Patient Register. Information on sickness absence periods and granted disability pensions were obtained from the Microdata for Analyses of Social Insurance of the Swedish Social Insurance Agency. Purchased medication data were obtained from the Prescribed Drug Register. Dates of death were obtained from the Cause of Death Register. Sociodemographic and labor market-related data were derived from the Longitudinal Database for Health Insurance and Labor Market Studies. The cohort entry date was the date of ADHD diagnosis, except for those who had their first diagnosis recorded before the age of 16 years because they entered the cohort at the age of 16 years. The follow-up ended at death, emigration, and end of data linkage (December 31, 2021), whichever occurred first. In analyses regarding sickness absence, the person was also censored after receiving disability pension.

Exposures were ADHD medications defined as Anatomical Therapeutic Chemical (ATC) code N06BA, clonidine (ATC code C02AC01), and guanfacine (ATC code C02AC02). Mixed amphetamine salts and combinations of amphetamine and dexamphetamine are coded as amphetamine, as per ATC category N06BA01. Concomitant use of 2 or more ADHD medications was defined as ADHD polytherapy and was analyzed as a separate category. Drug use periods (ie, when drug use started and ended) were constructed with the From Prescriptions to Drug Use Periods (PRE2DUP) method, a mathematical modeling method.^21^ On the basis of dispensing dates and dispensed amounts, the method calculates drug use periods by restricting the use with drug package–specific parameters that define clinically relevant upper and lower limits for daily dosage. The method takes into account individual purchasing regularity, stockpiling, and hospital care periods when drugs are provided by the caring unit and not recorded in the register.

Hospitalization for any psychiatric reason (psychiatric hospitalization) was defined as *ICD-10* codes F00 to F99. Suicide attempts or deaths due to suicide (suicidal behavior) were defined as *ICD-10 *codes X60 to X84 and codes Y10 to Y34 for hospitalizations and causes of death, and nonpsychiatric hospitalization was defined as any other code except *ICD-10 *codes F00 to F99. Work disability was defined as a period of sickness absence (>14 days) or granting of disability pension with any grade. In the analysis of work disability outcome, the individual was censored during sickness absence and disability pension periods because they were no longer at risk for this outcome.

For work disability analyses, additional requirements were set to ensure that individuals could be assumed to be in the labor market at cohort entry. We excluded persons who were already receiving disability pension at the cohort entry. We also required that to be included in the analyses, a person had been in the labor market at the end of the previous calendar year before cohort entry.

### Statistical Analysis

Data analysis was performed from November 2022 to August 2023. Within-individual Cox regression models were used where each individual forms their own stratum and acts as their own control. Follow-up time was reset to 0 after each outcome event to allow comparison of treatment periods within each individual. The design automatically adjusts for time-invariant factors, such as genetics and baseline severity of symptoms and baseline comorbidities. The analyses were adjusted for time-varying factors, which were temporal order of treatments (ie, which medication was used as first, second, third, and so forth, including also nonuse of ADHD medications), time since cohort entry, and time-varying use of psychotropic drugs, including antidepressants (ATC code N06A), anxiolytics (ATC code N05B), hypnotics (ATC code N05C), mood stabilizers (carbamazepine [ATC code N03AF01], valproic acid [ATC code N03AG01], and lamotrigine [ATC code N03AX09]), lithium (ATC code N05AN01), antipsychotics (ATC code N05A excluding lithium), and drugs for addictive disorders (ATC code N07B). Secondary analyses for the main outcome were by stratifying on sex and by age, defined as 16 to 29 years vs 30 to 65 years. Sensitivity analyses were performed by analyzing the data with a traditional between-individual Cox regression model. Statistical significance was defined as 2-sided *P* < .05. Data were analyzed with R statistical software version 4.3.2 (R Project for Statistical Computing).

## Results

The cohort included 221 714 individuals (mean [SD] age, 25.0 [11.2] years at cohort entry; 120 968 male participants [54.6%]) (Table). Because of the relatively young age distribution, the majority (135 199 individuals [61.0%]) had low educational status. In total, 125 164 (56.5%) had some psychiatric comorbidity, with anxiety or stress-related disorders (53 314 individuals [24.0%]) and depression and/or bipolar disorders (43 344 individuals [19.5%]) being the most common ones.

**Table.** Characteristics of the Study Population at Cohort Entry

Characteristic	Participants, No. (%) (N = 221 714)
Sex	
Female	100 746 (45.4)
Male	120 968 (54.6)
Age group, y	
16-29	156 836 (70.7)
≥30	64 856 (29.3)
Family status	
Youth living with parents	100 313 (46.6)
Single adult living without children	81 412 (36.7)
Single adult living with children	12 425 (5.8)
Married adult living with children	23 017 (10.7)
Married adult living without children	4547 (2.1)
Annual income from work, Sk^a^	
No income	116 973 (52.6)
1-100 000	57 390 (26.6)
100 001-200 000	16 078 (7.5)
200 001-300 000	15 044 (7.0)
300 001-400 000	9805 (4.5)
≥400 000	6424 (3.0)
Level of education, y	
0-9	135 199 (61.0)
10-12	61 427 (27.7)
≥12	25 088 (11.3)
Psychiatric comorbidities	
Anxiety and stress-related disorders	53 314 (24.0)
Depression or bipolar disorder	43 344 (19.5)
Autism spectrum disorders	25 033 (11.3)
Substance use disorders	20 604 (9.3)
Eating disorders	3185 (1.4)
Schizophrenia spectrum disorders	2500 (1.1)
Physical comorbidities	
Musculoskeletal diseases	15 309 (6.9)
Asthma	4220 (1.9)
Cardiovascular diseases	3916 (1.8)
Diabetes	2727 (1.2)
Any psychiatric comorbidity	
No	96 550 (43.5)
Yes	125 164 (56.5)
Any physical comorbidity	
No	118 739 (53.6)
Yes	102 975 (46.4)
No. of comorbidities	
0	58 583 (26.4)
1	68 173 (30.7)
2	49 832 (22.5)
>2	45 126 (20.4)

^a^ As of February 7, 2024, 1 Swedish krona = $0.095 US.

Methylphenidate was the most commonly used ADHD medication in this study, used by 68.5% of the cohort (151 837 individuals) during the follow-up period (eTable in Supplement 1). Lisdexamphetamine was the second most common medication (78 106 individuals [35.2%]), followed by polytherapy of ADHD medications (60 102 individuals [27.1%]) and atomoxetine (34 631 individuals [15.6%]).

**Figure 1.**
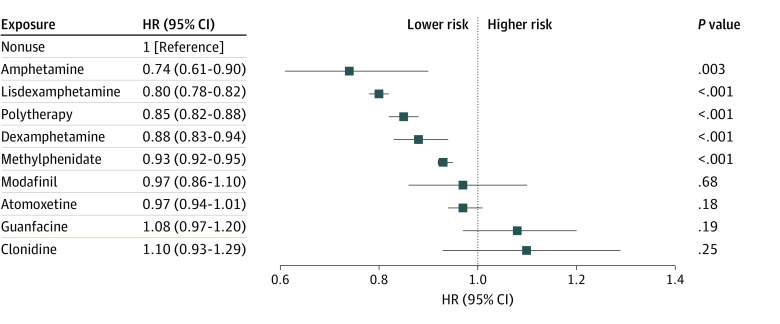
Psychiatric Hospitalization Forest plot shows adjusted hazard ratios (HRs) and 95% CIs for risk of psychiatric hospitalization associated with specific attention-deficit/hyperactivity disorder drugs (vs no use of such drugs) in within-individual design.

During the 15 years of follow-up (mean [SD], 7.0 [4.7] years; median [IQR], 6.3 [2.9-10.5] years), 56 704 individuals (25.6%) experienced psychiatric hospitalization. Medications associated with a decreased risk of psychiatric hospitalization were amphetamine (aHR, 0.74; 95% CI, 0.61-0.90), lisdexamphetamine (aHR, 0.80; 95% CI, 0.78-0.82), ADHD polytherapy (aHR, 0.85; 95% CI, 0.82-0.88), dexamphetamine (aHR, 0.88; 95% CI, 0.83-0.94), and methylphenidate (aHR, 0.93; 95% CI, 0.92-0.95), whereas no association was found for modafinil, atomoxetine, clonidine, and guanfacine (Figure 1 and eTable in Supplement 1). When stratified by age at baseline, lisdexamphetamine was associated with decreased risk of psychiatric hospitalization both among adolescents and young adults (aHR, 0.81; 95% CI, 0.78-0.83) and adults aged 30 years and older (aHR, 0.78; 95% CI, 0.74-0.82) (eFigures 1 and 2 in Supplement 1). The results for amphetamine and dexamphetamine varied more, with dexamphetamine associated with decreased risk among adolescents and young adults but not among those aged 30 years and older, whereas the results for amphetamine were the opposite. Methylphenidate was associated with a decreased risk in adolescents and young adults (aHR, 0.91; 95% CI, 0.89-0.93) but less so among older ones (aHR, 0.97; 95% CI, 0.94-0.99). Atomoxetine was associated with decreased risk among female participants (aHR, 0.94; 95% CI, 0.89-0.99) but not among male participants (eFigures 3 and 4 in Supplement 1). Other small differences in the results of less common medications (eg, amphetamine and dexamphetamine) are likely related to lack of statistical power. The point estimates for lisdexamphetamine (0.80 in female participants vs 0.80 in male participants) and methylphenidate (0.93 in female participants vs 0.94 in male participants) were very similar between female and male participants, indicating that ADHD medications are equally effective in reducing the risk of psychiatric hospitalization between the sexes. In between-individual analyses of the main outcome, the rank order was similar to that in the main analyses, although point estimates tended to be somewhat lower than in the within-individual analyses (eFigure 5 in Supplement 1).

**Figure 2.**
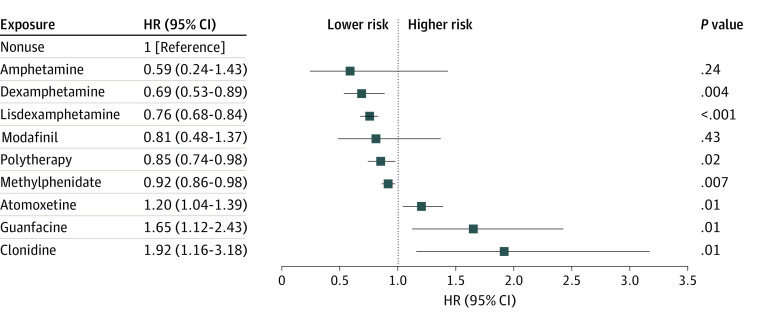
Suicide Attempt and/or Death Forest plot shows adjusted hazard ratios (HRs) and 95% CIs for risk of suicide attempt and/or death associated with specific attention-deficit/hyperactivity disorder drugs (vs no use of such drugs) in within-individual design. Drugs with fewer than 30 events are not shown.

Suicidal behavior occurred for 10 668 individuals (4.8%) during the follow-up. Use of dexamphetamine (aHR, 0.69; 95% CI, 0.53-0.89), lisdexamphetamine (aHR, 0.76; 95% CI, 0.68-0.84), polytherapy (aHR, 0.85; 95% CI, 0.74-0.98), and methylphenidate (aHR, 0.92; 95% CI, 0.86-0.98) were associated with a decreased risk of suicidal behavior, whereas atomoxetine was associated with an increased risk (aHR, 1.20; 95% CI, 1.04-1.39) (Figure 2). In between-individual analyses, all medications except guanfacine and clonidine were associated with a decreased risk of suicidal behavior (eFigure 6 in Supplement 1).

**Figure 3.**
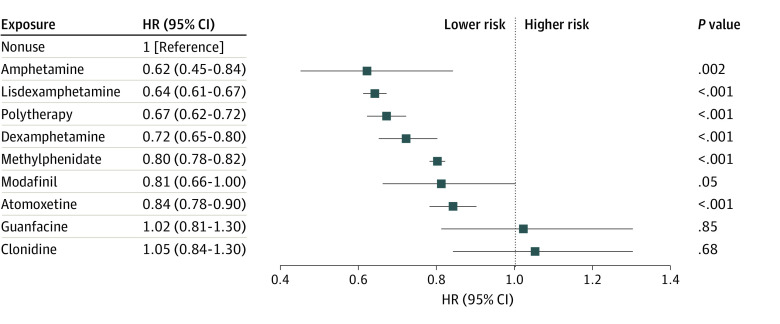
Nonpsychiatric Hospitalization Forest plot shows adjusted hazard ratios (HRs) and 95% CIs for risk of nonpsychiatric hospitalization associated with specific attention-deficit/hyperactivity disorder drugs (vs no use of such drugs) in within-individual design.

Nonpsychiatric hospitalization happened for 58 352 individuals (26.3%), and it was used as a proxy for safety. However, we did not observe evidence of increased risk of nonpsychiatric hospitalization. In fact, the use of amphetamine (aHR, 0.62; 95% CI, 0.45-0.84), lisdexamphetamine (aHR, 0.64; 95% CI, 0.61-0.67), polytherapy (aHR, 0.67; 95% CI, 0.62-0.72), dexamphetamine (aHR, 0.72; 95% CI, 0.65-0.80), methylphenidate (aHR, 0.80; 95% CI, 0.78-0.82), and atomoxetine (aHR, 0.84; 95% CI, 0.78-0.90) were associated with decreased risk of nonpsychiatric hospitalization (Figure 3). The results of between-individual analyses were in line with the results of the within-individual analyses (eFigure 7 in Supplement 1).

**Figure 4.**
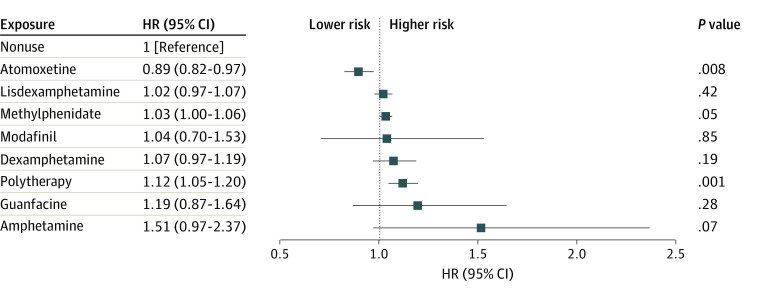
Work Disability Forest plot shows adjusted hazard ratios (HRs) and 95% CIs for risk of work disability, measured as sickness absence and/or disability pension, associated with specific attention-deficit/hyperactivity disorder drugs (vs no use of such drugs) in within-individual design. The analyses included 189 380 persons who were not receiving a disability pension at the cohort entry. Drugs with fewer than 30 events are not shown.

In the analyses of work disability, 189 380 individuals could be included because they were not receiving a disability pension at the cohort entry and fulfilled the minimum requirement for presumably being in the labor market. Of them, the work disability event was registered for 56 835 individuals (30.0%) during the mean (SD) follow-up of 6.0 (4.1) years (median [IQR], 5.5 [2.6-9.0] years). Atomoxetine was associated with a slightly decreased risk (aHR, 0.89; 95% CI, 0.82-0.97), and polytherapy was associated with an increased risk (aHR, 1.12; 95% CI, 1.05-1.20) of work disability, whereas the results for other medications were not significant (Figure 4). Between-individual analyses showed somewhat decreased risk associated with the most common medications, with aHRs of 0.90 to 0.93 (eFigure 8 in Supplement 1). In within-individual analyses, atomoxetine (aHR, 0.82; 95% CI, 0.73-0.92) and methylphenidate (aHR, 0.88; 95% CI, 0.85-0.92) were associated with a decreased risk of work disability among adolescents and young adults aged 16 to 29 years but not among those aged 30 years or older (eFigures 9 and 10 in Supplement 1). When stratified by sex, atomoxetine was associated with decreased risk of work disability only among male participants (aHR, 0.85; 95% CI, 0.75-0.97) and not among female participants (aHR, 0.92; 95% CI, 0.83-1.03) (eFigure 11 and 12 in Supplement 1).

## Discussion

This nationwide cohort study of 221 714 adolescents and adults with ADHD found that compared with periods when medications were not used, the use of ADHD medications, especially the stimulants lisdexamphetamine, dexamphetamine, amphetamine, and methylphenidate, was associated with a decreased risk of psychiatric hospitalization, suicidal behavior, and nonpsychiatric hospitalizations; in contrast, nonstimulant atomoxetine use was associated only with decreased risk of work disability, especially among adolescents and young adults. The overall effect on psychiatric outcomes was beneficial, and, similarly, we found a decreased (rather than increased) risk of nonpsychiatric hospitalizations.

This study is one of the first to assess the risk of outcomes among persons with ADHD associated with specific ADHD medications, because most previous studies have investigated ADHD medications as a group^10,12^ or have focused on stimulants only^8,9,11^ or grouped medications as stimulants vs nonstimulants.^7,22^ In long-term follow-up of up to 15 years (mean, 7 years), amphetamine derivatives, especially lisdexamphetamine but also amphetamine and dexamphetamine, were associated with decreased risk of both psychiatric hospitalization and suicidal behavior. Although concerns have been raised about the potential of amphetamines and methylphenidate for increasing the risk of adverse psychiatric outcomes, such as psychosis and mania,^15,16^ our results show that overall the net effect on psychiatric outcomes is positive. This is in line with several previous studies showing decreased risk of suicidal behavior^7^ and development of substance use disorder,^8^ as well as lower rates of occurrence and reoccurrence of depression^9^ during ADHD drug use. There were some differences in the results when stratified by age, which can be at least partially related to statistical power issues, mainly caused by less frequent use of amphetamine among adolescents and young adults. Our results showed consistently that lisdexamphetamine was associated with decreased risk of psychiatric hospitalization and suicidal behavior, which is well in line with the results from studies concerning individuals with borderline personality disorder,^23,24^ as well as individuals with amphetamine use disorder.^25^

Methylphenidate was associated with decreased risk in adolescents and young adults (aHR, 0.91; 95% CI, 0.89-0.93) but less so among older individuals (aHR, 0.97; 95% CI, 0.94-0.99). This is in line with the findings of a meta-analysis of short-term RCTs,^5^ which recommended methylphenidate use for children and adolescents and amphetamines for adults, although the age ranges differed from those in the current study. However, it seems that methylphenidate either is less effective among those aged 30 years or older or there is some kind of initial effect that diminishes with longer-term use, age, or course of illness, because methylphenidate is usually prescribed as the first-line medication. In this study, we cannot separate which of these time axes is more relevant. However, for all outcomes and strata studied, lisdexamphetamine had aHRs less than or equal to those for methylphenidate.

Associations of ADHD drug use with nonpsychiatric hospitalizations were mainly studied as a potential indicator of safety problems (eg, due to cardiovascular adverse effects or an increased risk of seizures).^14,26^ However, none of the ADHD drugs was associated with increased risk of nonpsychiatric hospitalization, and amphetamine, lisdexamphetamine, polytherapy, dexamphetamine, methylphenidate, and atomoxetine were associated with decreased risk. This is in line with a previous study^26^ showing decreased rather than increased risk of acute seizures among people with ADHD and comorbid epilepsy. This may be related to the fact that ADHD medication use tends to decrease the risk of unintentional injuries.^11^ We investigated only hospitalizations, referring to severe events, and, thus, cannot rule out the possibility that ADHD medication use can be associated with less severe events.

The results for the nonstimulant medication atomoxetine were mixed and less clear than those for stimulants. Atomoxetine showed no association with psychiatric hospitalization, and but was associated with a 20% increased risk of suicidal behavior (aHR, 1.20), a 16% decreased risk of nonpsychiatric hospitalization (aHR, 0.84), and an 11% decreased risk of work disability (aHR, 0.89). This finding is in line with previous studies reporting generally less favorable outcomes for nonstimulants than for stimulants.^7,22^ Atomoxetine was used by 15.6% of the study population, which made it more frequent than, for example, amphetamine and dexamphetamine, which showed decreased risk of several outcomes. Because atomoxetine is a nonstimulant drug, it is likely selectively prescribed for persons who have contraindications and for those who have experienced or who fear adverse effects of stimulants.

The results for work disability were somewhat unexpected, because we did not notice a decreased risk associated with any of the stimulant medications (except methylphenidate among adolescents and young adults), although those drugs are associated with a decreased risk of hospitalizations, which automatically lead to and describe a state of not being able to work. Atomoxetine, however, was associated with a decreased risk of work disability, especially among younger people, which may imply that perhaps atomoxetine is prescribed for patients with less-severe ADHD who have better chances of participating in working life. Previous studies by us^27,28^ and others^18,29^ have shown that participation in the labor market is very difficult for people with ADHD, and they may already have reached the point of exclusion from the labor market before the start of follow-up for this study. The stimulant medication use may also be connected with a higher level of adverse effects, which might hamper the ability to work at a higher grade than for nonstimulants. Even if confounding by indication might be avoided through the design of this study, we cannot know the clinical reasons, and situations for choosing a particular medication and stimulant medication may be channeled to more severe cases.

### Strengths and Limitations

Strengths of this study include the use of nationwide data from a large and representative cohort of persons with ADHD and a long follow-up time. Drug dispensing was processed into drug use periods with a validated PRE2DUP method, resulting in reliable estimates of periods when drugs were used vs not used.^30^ The PRE2DUP method also enabled us to study associations by drug level and by separating polytherapy of ADHD medication use from monotherapy use. We used within-individual models to minimize selection bias—namely, certain treatments are selected on the basis of patient characteristics and symptoms. The temporal order of treatments was adjusted for in the analyses, which decreases the impact of certain medications, such as methylphenidate being the first-line pharmacotherapy, whereas certain other treatments are used later in the course of illness or when first-line treatments have not been successful or tolerable.

Limitations of this study are related to the nature of the data sources used—namely, nationwide registers and the consequent lack of detailed clinical data. These include the type and severity of symptoms and concomitant symptoms related to other comorbid psychiatric conditions, which were common in the study cohort. The symptomatic phases of other psychiatric disorders were adjusted for, in part, when we adjusted for psychotropic medication use. We also lacked data on nonpharmacological treatments, including psychoeducation and psychological therapies, and whether patients actually had access to these treatments. In the analyses of work disability, data sources did not enable accurate and time-varying identification of those who are in the labor market and are at risk for this outcome (eg, due to shorter term events such as parental leave or going back to studies). This may have impacted the results of work disability by diluting the estimates toward the null values. In addition, we lacked data on sickness absence periods of less than 14 days. Suicide attempts in general are underreported, and, thus, our analyses represent attempts that have led to hospital admission. The results of this study are generalizable to health care systems resembling that of Sweden (ie, those that provide equal access to services for all residents). The study population may not represent the full spectrum of population diversity as observed across the globe.

## Conclusions

In this cohort study of adolescents and adults with ADHD, the use of medications for ADHD, especially lisdexamphetamine and other stimulants, was associated with decreased risk of psychiatric hospitalizations, suicidal behavior, and nonpsychiatric hospitalizations during periods when they were used compared with periods when ADHD medication was not used. Nonstimulant atomoxetine use was associated with decreased risk of work disability. Considering the high prevalence of psychiatric comorbidity in persons with ADHD, these results suggest that ADHD medication use can reduce morbidity in adolescents and adults with ADHD.

## Notes

Supplement 1. **eFigure 1.** Risk of Psychiatric Hospitalization Associated With Specific ADHD Drugs (Compared to Nonuse of ADHD Drugs) in Within-Individual Design Among Those Aged 16-29 Years at Baseline **eFigure 2.** Risk of Psychiatric Hospitalization Associated With Specific ADHD Drugs (Compared to Nonuse of ADHD Drugs) in Within-Individual Design Among Those Aged ≥30 Years at Baseline **eFigure 3.** Risk of Psychiatric Hospitalization Associated With Specific ADHD Drugs (Compared to Nonuse of ADHD Drugs) in Within-Individual Design Among Women **eFigure 4.** Risk of Psychiatric Hospitalization Associated With Specific ADHD Drugs (Compared to Nonuse of ADHD Drugs) in Within-Individual Design Among Men **eFigure 5.** Risk of Psychiatric Hospitalization Associated With Specific ADHD Drugs (Compared to Nonuse of ADHD Drugs) in Traditional Between-Individual Design **eFigure 6.** Risk of Psychiatric Suicide Attempt/Death Associated With Specific ADHD Drugs (Compared to Nonuse of ADHD Drugs) in Traditional Between-Individual Design **eFigure 7.** Risk of Nonpsychiatric Hospitalization Associated With Specific ADHD Drugs (Compared to Nonuse of ADHD Drugs) in Traditional Between-Individual Design **eFigure 8.** Risk of Work Disability Associated With Specific ADHD Drugs (Compared to Nonuse of ADHD Drugs) in Traditional Between-Individual Design **eFigure 9.** Risk of Work Disability Associated With Specific ADHD Drugs (Compared to Nonuse of ADHD Drugs) in Within-Individual Design Among Those Aged 16-29 Years at Baseline **eFigure 10.** Risk of Work Disability Associated With Specific ADHD Drugs (Compared to Nonuse of ADHD Drugs) in Within-Individual Design Among Those Aged ≥30 Years at Baseline **eFigure 11.** Risk of Work Disability Associated With Specific ADHD Drugs (Compared to Nonuse of ADHD Drugs) in Within-Individual Design Among Women **eFigure 12.** Risk of Work Disability Associated With Specific ADHD Drugs (Compared to Nonuse of ADHD Drugs) in Within-Individual Design Among Men **eTable.** Number of Users, Person-Years and Events for Different Outcomes During the Follow-Up in the Cohort of Persons With ADHD (N=221,714)

Supplement 2.
Data Sharing Statement
