# Supplementary material for: Attention-Deficit/Hyperactivity Disorder Medications and Work Disability and Mental Health Outcomes
Source: JAMA Netw Open. 2024 Mar 20;7(3):e242859. doi: 10.1001/jamanetworkopen.2024.2859 (PMC10955386; doi:10.1001/jamanetworkopen.2024.2859)
Supplement: Supplement 1. — eFigure 1. Risk of Psychiatric Hospitalization Associated With Specific ADHD Drugs (Compared to Nonuse of ADHD Drugs) in Within-Individual Design Among Those Aged 16-29 Years at Baseline eFigure 2. Risk of Psychiatric Hospitalization Associated With Specific ADHD Drugs (Compared to Nonuse of ADHD Drugs) in Within-Individual Design Among Those Aged ≥30 Years at Baseline eFigure 3. Risk of Psychiatric Hospitalization Associated With Specific ADHD Drugs (Compared to Nonuse of ADHD Drugs) in Within-Individual Design Among Women eFigure 4. Risk of Psychiatric Hospitalization Associated With Specific ADHD Drugs (Compared to Nonuse of ADHD Drugs) in Within-Individual Design Among Men eFigure 5. Risk of Psychiatric Hospitalization Associated With Specific ADHD Drugs (Compared to Nonuse of ADHD Drugs) in Traditional Between-Individual Design eFigure 6. Risk of Psychiatric Suicide Attempt/Death Associated With Specific ADHD Drugs (Compared to Nonuse of ADHD Drugs) in Traditional Between-Individual Design eFigure 7. Risk of Nonpsychiatric Hospitalization Associated With Specific ADHD Drugs (Compared to Nonuse of ADHD Drugs) in Traditional Between-Individual Design eFigure 8. Risk of Work Disability Associated With Specific ADHD Drugs (Compared to Nonuse of ADHD Drugs) in Traditional Between-Individual Design eFigure 9. Risk of Work Disability Associated With Specific ADHD Drugs (Compared to Nonuse of ADHD Drugs) in Within-Individual Design Among Those Aged 16-29 Years at Baseline eFigure 10. Risk of Work Disability Associated With Specific ADHD Drugs (Compared to Nonuse of ADHD Drugs) in Within-Individual Design Among Those Aged ≥30 Years at Baseline eFigure 11. Risk of Work Disability Associated With Specific ADHD Drugs (Compared to Nonuse of ADHD Drugs) in Within-Individual Design Among Women eFigure 12. Risk of Work Disability Associated With Specific ADHD Drugs (Compared to Nonuse of ADHD Drugs) in Within-Individual Design Among Men eTable. Number of Users, Person-Years an [file jamanetwopen-e242859-s001.pdf]

Supplemental Online Content

Taipale H, Bergström J, Gèmes K, et al. Attention-deficit/hyperactivity disorder medications and work disability and mental health outcomes. *JAMA Netw Open*. 2024;7(3):e242859. doi:10.1001/jamanetworkopen.2024.2859

eFigure 1. Risk of Psychiatric Hospitalization Associated With Specific ADHD Drugs (Compared to Nonuse of ADHD Drugs) in Within-Individual Design Among Those Aged 16-29 Years at Baseline

eFigure 2. Risk of Psychiatric Hospitalization Associated With Specific ADHD Drugs (Compared to Nonuse of ADHD Drugs) in Within-Individual Design Among Those Aged ≥30 Years at Baseline

eFigure 3. Risk of Psychiatric Hospitalization Associated With Specific ADHD Drugs (Compared to Nonuse of ADHD Drugs) in Within-Individual Design Among Women

eFigure 4. Risk of Psychiatric Hospitalization Associated With Specific ADHD Drugs (Compared to Nonuse of ADHD Drugs) in Within-Individual Design Among Men

eFigure 5. Risk of Psychiatric Hospitalization Associated With Specific ADHD Drugs (Compared to Nonuse of ADHD Drugs) in Traditional Between-Individual Design

eFigure 6. Risk of Psychiatric Suicide Attempt/Death Associated With Specific ADHD Drugs (Compared to Nonuse of ADHD Drugs) in Traditional Between-Individual Design

eFigure 7. Risk of Nonpsychiatric Hospitalization Associated With Specific ADHD Drugs (Compared to Nonuse of ADHD Drugs) in Traditional Between-Individual Design

eFigure 8. Risk of Work Disability Associated With Specific ADHD Drugs (Compared to Nonuse of ADHD Drugs) in Traditional Between-Individual Design

eFigure 9. Risk of Work Disability Associated With Specific ADHD Drugs (Compared to Nonuse of ADHD Drugs) in Within-Individual Design Among Those Aged 16-29 Years at Baseline

eFigure 10. Risk of Work Disability Associated With Specific ADHD Drugs (Compared to Nonuse of ADHD Drugs) in Within-Individual Design Among Those Aged ≥30 Years at Baseline

eFigure 11. Risk of Work Disability Associated With Specific ADHD Drugs (Compared to Nonuse of ADHD Drugs) in Within-Individual Design Among Women

eFigure 12. Risk of Work Disability Associated With Specific ADHD Drugs (Compared to Nonuse of ADHD Drugs) in Within-Individual Design Among Men

eTable. Number of Users, Person-Years and Events for Different Outcomes During the Follow-Up in the Cohort of Persons With ADHD (N=221,714)

This supplemental material has been provided by the authors to give readers additional information about their work.

© 2024 Taipale H et al. *JAMA Network Open*.

eFigure 1. Risk of Psychiatric Hospitalization Associated With Specific ADHD Drugs (Compared to Nonuse of ADHD Drugs) in Within-Individual Design Among Those Aged 16-29 Years at Baseline

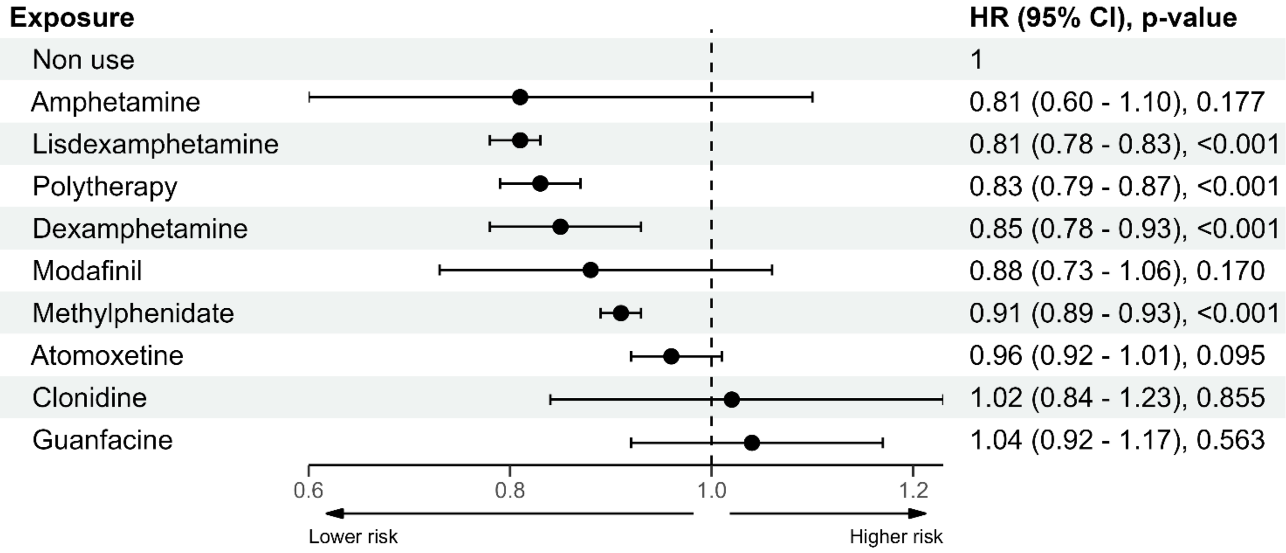

eFigure 2. Risk of Psychiatric Hospitalization Associated With Specific ADHD Drugs (Compared to Nonuse of ADHD Drugs) in Within-Individual Design Among Those Aged ≥30 Years at Baseline

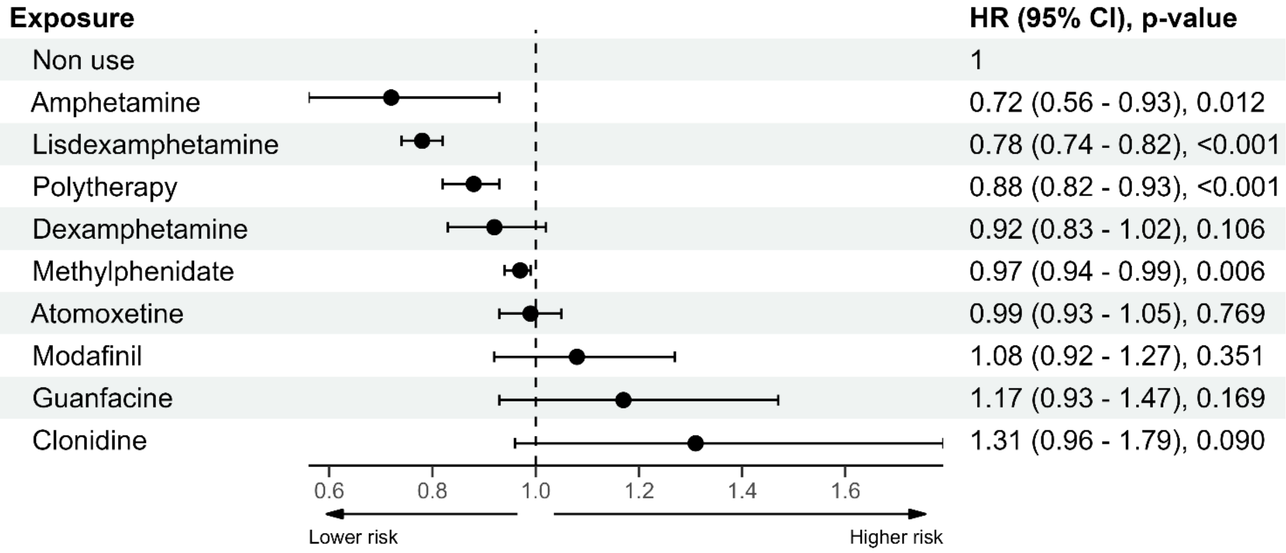

© 2024 Taipale H et al. *JAMA Network Open*.

eFigure 3. Risk of Psychiatric Hospitalization Associated With Specific ADHD Drugs (Compared to Nonuse of ADHD Drugs) in Within-Individual Design Among Women

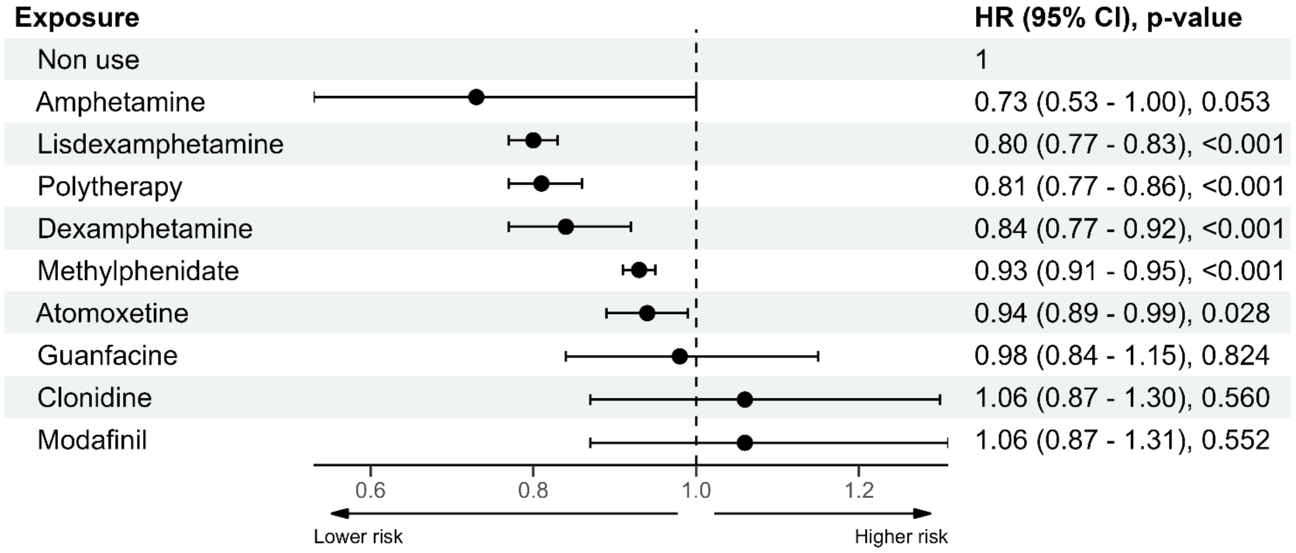

eFigure 4. Risk of Psychiatric Hospitalization Associated With Specific ADHD Drugs (Compared to Nonuse of ADHD Drugs) in Within-Individual Design Among Men

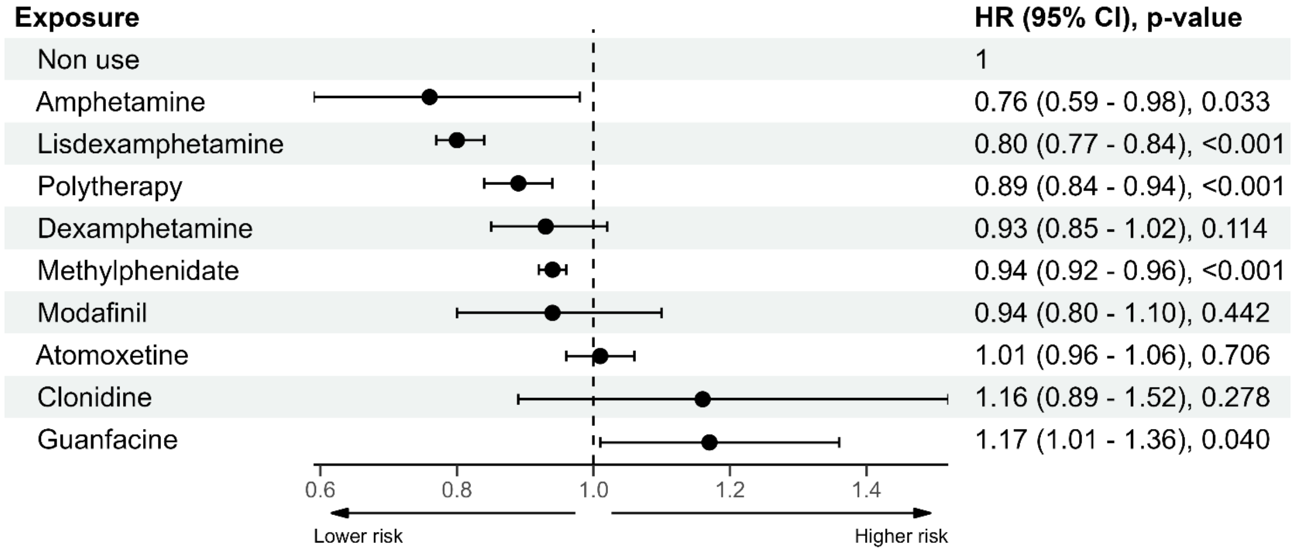

© 2024 Taipale H et al. *JAMA Network Open*.

eFigure 5. Risk of Psychiatric Hospitalization Associated With Specific ADHD Drugs (Compared to Nonuse of ADHD Drugs) in Traditional Between-Individual Design.

Adjusted for baseline covariates including age, sex, income, number of unemployment days, country of birth, education, marital status, living area, number of diagnosed physical diseases (cardiovascular diseseaes, musculoskeletal diseases, diabetes, asthma), the number of psychiatric comorbities (anxiety and stress-related disorders, depression or bipolar disorder, autism-spectrum disorder, substance use disorder, eating disorder, schizophrenia-spectrum disorders), and for time-varying use of antidepressants (ATC N06A), anxiolytics (N05B), hypnotics (N06C), mood stabilizers (valproic acid, carbamazepine, lamotrigine), lithium, antipsychotics (N05A), drugs for addictions (N07B), temporal orders of ADHD medications used and time since cohort entry.

Exposure	HR (95% CI), p-value
Non use	1
Lisdexamphetamine	0.66 (0.64 - 0.68), <0.001
Polytherapy	0.71 (0.69 - 0.74), <0.001
Amphetamine	0.72 (0.61 - 0.84), <0.001
Dexamphetamine	0.73 (0.69 - 0.77), <0.001
Atomoxetine	0.80 (0.77 - 0.84), <0.001
Methylphenidate	0.82 (0.81 - 0.83), <0.001
Modafinil	0.95 (0.84 - 1.09), 0.491
Guanfacine	0.96 (0.86 - 1.06), 0.424
Clonidine	1.28 (1.04 - 1.57), 0.020

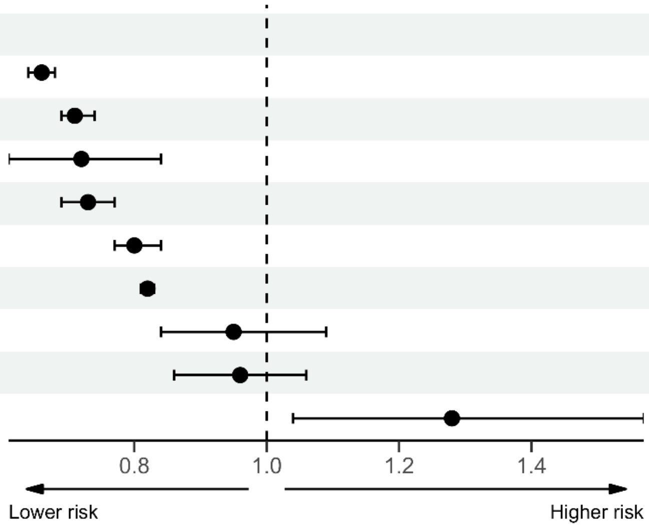

eFigure 6. Risk of Psychiatric Suicide Attempt/Death Associated With Specific ADHD Drugs (Compared to Nonuse of ADHD Drugs) in Traditional Between-Individual Design. Adjusted for the same factors as eFigure 5.

Exposure	HR (95% CI), p-value
Non use	1
Amphetamine	0.56 (0.35 - 0.91), 0.019
Dexamphetamine	0.59 (0.45 - 0.77), <0.001
Lisdexamphetamine	0.60 (0.55 - 0.64), <0.001
Polytherapy	0.64 (0.56 - 0.72), <0.001
Modafinil	0.67 (0.47 - 0.96), 0.027
Atomoxetine	0.74 (0.67 - 0.83), <0.001
Methylphenidate	0.78 (0.74 - 0.82), <0.001
Guanfacine	1.00 (0.76 - 1.31), 0.977
Clonidine	1.39 (0.86 - 2.23), 0.174

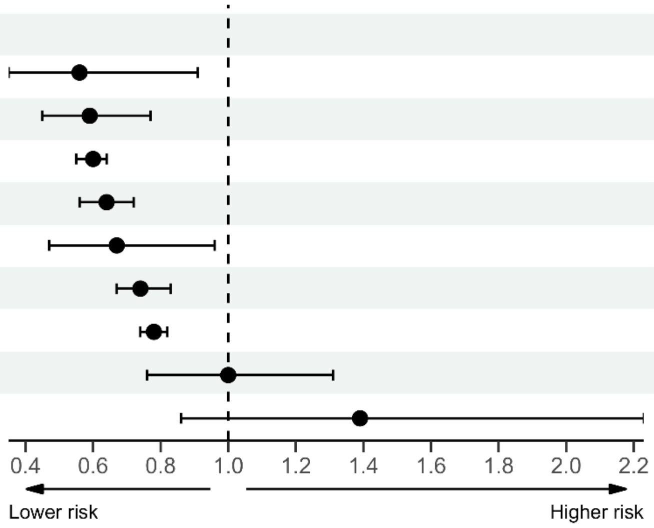

© 2024 Taipale H et al. *JAMA Network Open*.

eFigure 7. Risk of Nonpsychiatric Hospitalization Associated With Specific ADHD Drugs (Compared to Nonuse of ADHD Drugs) in Traditional Between-Individual Design. Adjusted for the same factors as eFigure 5.

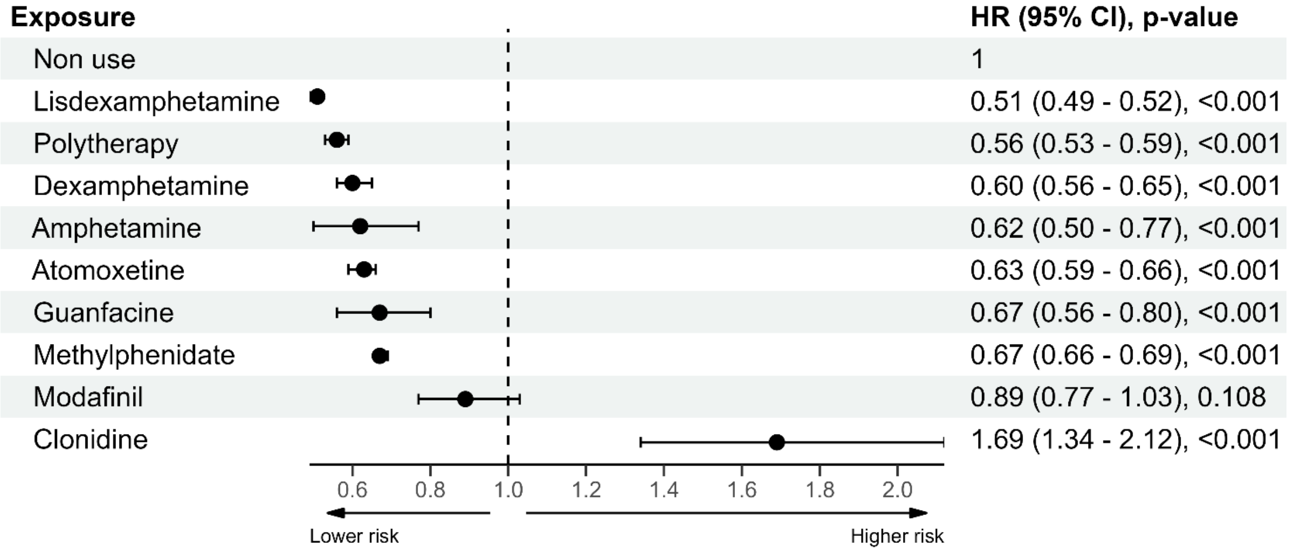

eFigure 8. Risk of Work Disability Associated With Specific ADHD Drugs (Compared to Nonuse of ADHD Drugs) in Traditional Between-Individual Design. Adjusted for the same factors as eFigure 5.

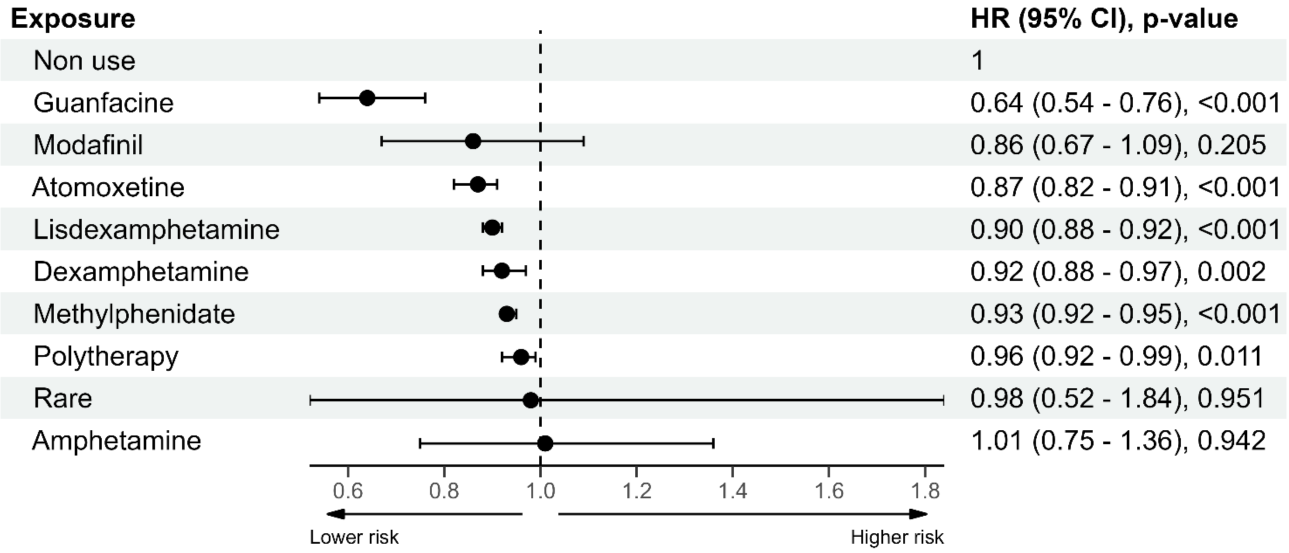

© 2024 Taipale H et al. *JAMA Network Open*.

eFigure 9. Risk of Work Disability Associated With Specific ADHD Drugs (Compared to Nonuse of ADHD Drugs) in Within-Individual Design Among Those Aged 16-29 Years at Baseline. “Rare” category includes drugs with <30 events.

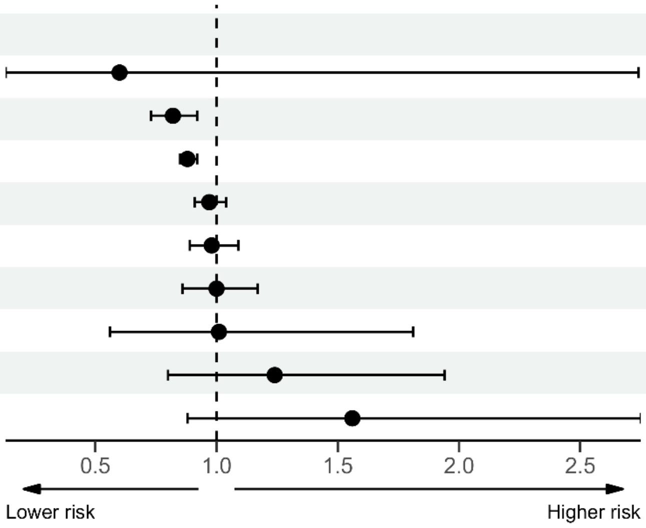

Exposure	HR (95% CI), p-value
Non use	1
Rare	0.60 (0.13 - 2.74), 0.511
Atomoxetine	0.82 (0.73 - 0.92), <0.001
Methylphenidate	0.88 (0.85 - 0.92), <0.001
Lisdexamphetamine	0.97 (0.91 - 1.04), 0.379
Polytherapy	0.98 (0.89 - 1.09), 0.741
Dexamphetamine	1.00 (0.86 - 1.17), 0.980
Modafinil	1.01 (0.56 - 1.81), 0.972
Guanfacine	1.24 (0.80 - 1.94), 0.340
Amphetamine	1.56 (0.88 - 2.75), 0.125

eFigure 10. Risk of Work Disability Associated With Specific ADHD Drugs (Compared to Nonuse of ADHD Drugs) in Within-Individual Design Among Those Aged ≥30 Years at Baseline. “Rare” category includes drugs with <30 events.

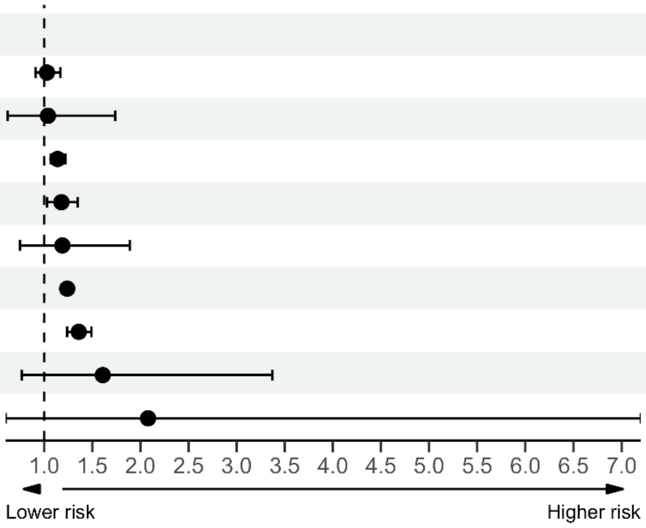

Exposure	HR (95% CI), p-value
Non use	1
Atomoxetine	1.03 (0.91 - 1.17), 0.618
Modafinil	1.04 (0.62 - 1.74), 0.882
Lisdexamphetamine	1.14 (1.07 - 1.22), <0.001
Dexamphetamine	1.18 (1.03 - 1.35), 0.020
Guanfacine	1.19 (0.75 - 1.89), 0.468
Methylphenidate	1.24 (1.18 - 1.29), <0.001
Polytherapy	1.36 (1.24 - 1.49), <0.001
Amphetamine	1.61 (0.77 - 3.37), 0.207
Rare	2.08 (0.60 - 7.20), 0.246

© 2024 Taipale H et al. *JAMA Network Open*.

eFigure 11. Risk of Work Disability Associated With Specific ADHD Drugs (Compared to Nonuse of ADHD Drugs) in Within-Individual Design Among Women. “Rare” category includes drugs with <30 events.

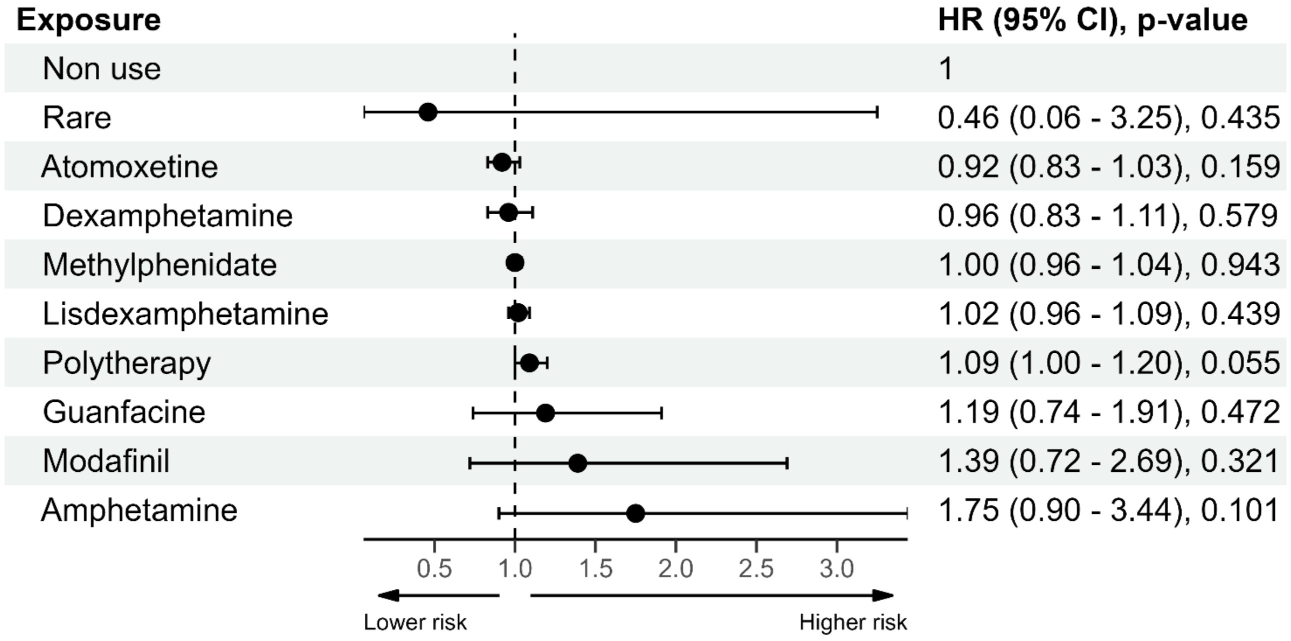

eFigure 12. Risk of Work Disability Associated With Specific ADHD Drugs (Compared to Nonuse of ADHD Drugs) in Within-Individual Design Among Men. “Rare” category includes drugs with <30 events.

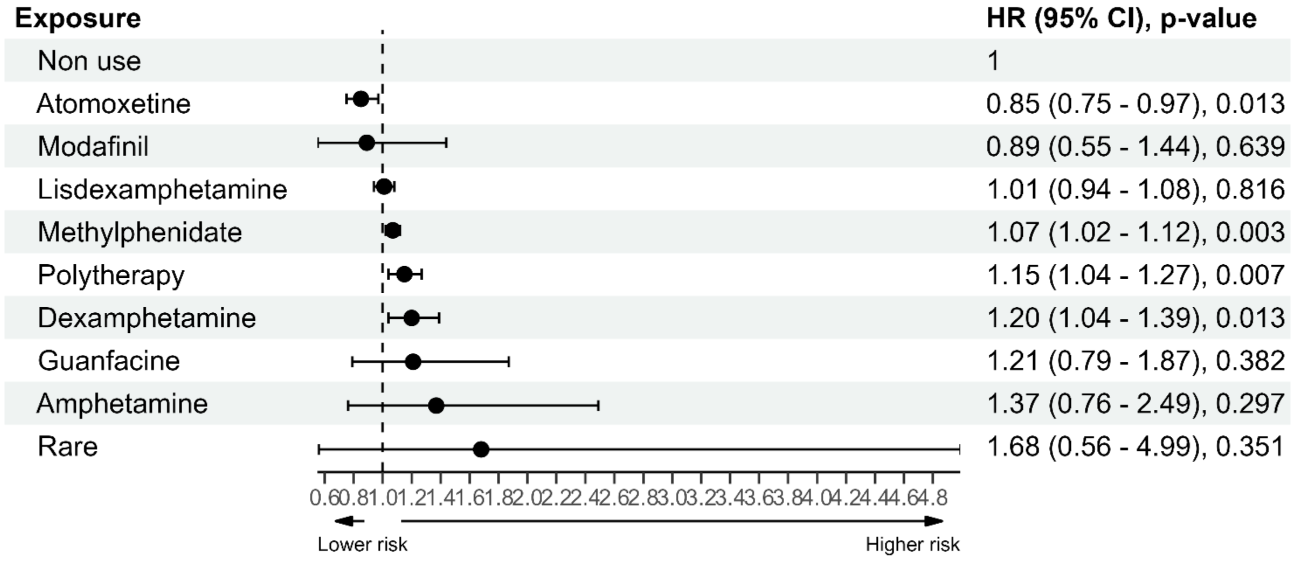

© 2024 Taipale H et al. *JAMA Network Open*.

eTable. Number of Users, Person-Years and Events for Different Outcomes During the Follow-Up in the Cohort of Persons With ADHD (N=221,714).

			Psychiatric hospitalization	Suicide attempt/death	Non-psychiatric hospitalization	Work disability
	Users, %(N)	Person-years	Events, N	Events, N	Events, N	Events, N
Non-use	92.1 (204270)	1012700	158914	12825	100652	74210
Methylphenidate	68.5 (151837)	321803	45136	3988	20965	24693
Dexamphetamine	5.2 (11468)	18367	2539	207	1316	1802
Lisdexamphetamine	35.2 (78106)	108689	13297	1332	5951	11297
Polytherapy	27.1 (60102)	36695	5545	554	2324	3842
Guanfacine	2.5 (5502)	3161	705	82	207	138
Atomoxetine	15.6 (34631)	30558	5183	501	1988	1989
Modafinil	0.7 (1613)	1646	444	31	200	81
Clonidine	0.3 (577)	517	256	37	168	NA
Amphetamine	0.3 (558)	1964	268	19	129	78

NA: these exposures were not analyzed nor shown here due to sparsity of events.

© 2024 Taipale H et al. *JAMA Network Open*.
