# Supplementary material for: Attention-Deficit/Hyperactivity Disorder Medications and Work Disability and Mental Health Outcomes
Source: JAMA Netw Open. 2024 Mar 20;7(3):e242859. doi: 10.1001/jamanetworkopen.2024.2859 (PMC10955386; doi:10.1001/jamanetworkopen.2024.2859)
Supplement: Supplement 2. — Data Sharing Statement [file jamanetwopen-e242859-s002.pdf]

Data Sharing Statement

Taipale. Attention-Deficit/Hyperactivity Disorder Medications and Work Disability and Mental Health Outcomes. *JAMA Netw Open*. Published March 20, 2024.
doi:10.1001/jamanetworkopen.2024.2859

Data
Data available: No

Additional Information
Explanation for why data not available: The project utilized data from the REWHARD consortium, supported by the Swedish Research Council (VR grant number. 2017-00624). These data cannot be made publicly available due to privacy regulations. According to the General Data Protection Regulation, the Swedish law SFS 2018:218, the Swedish Data Protection Act, the Swedish Ethical Review Act, and the Public Access to Information and Secrecy Act, these types of sensitive data can only be made available for specific purposes, including research, that meet the criteria for access to these type of sensitive and confidential data as determined by a legal review.
